# Compound heterozygous *CACNA1H* mutations associated with severe congenital amyotrophy

**DOI:** 10.1080/19336950.2019.1614415

**Published:** 2019-05-09

**Authors:** Melissa T. Carter, Hugh J. McMillan, Andriy Tomin, Norbert Weiss

**Affiliations:** a Children’s Hospital of Eastern Ontario Research Institute, University of Ottawa, Ottawa, Ontario, Canada; b Institute of Organic Chemistry and Biochemistry, Czech Academy of Sciences, Prague, Czech Republic

**Keywords:** Congenital amyotrophy, CACNA1H, mutations, calcium channel, Ca_v_3.2 channel, T-type channel

## Abstract

Neuromuscular disorders encompass a wide range of conditions often associated with a genetic component. In the present study, we report a patient with severe infantile-onset amyotrophy in whom two compound heterozygous variants in the gene *CACNA1H* encoding for Ca_v_3.2 T-type calcium channels were identified. Functional analysis of Ca_v_3.2 variants revealed several alterations of the gating properties of the channel that were in general consistent with a loss-of-channel function. Taken together, these findings suggest that severe congenital amyoplasia may be related to *CACNA1H* and would represent a new phenotype associated with mutations in this gene.

## Introduction

Neuromuscular disorders (NMD) encompass a wide range of conditions including amyotrophic lateral sclerosis (ALS), congenital muscular dystrophies and myopathies, Duchenne muscular dystrophy, and spinal muscular atrophy. They are characterized by progressive muscle degeneration and weakness that primarily or secondarily impair skeletal muscles and their innervation. The onset of NMD ranges from *in utero* to old age, but primarily affect infants, children, and teenagers [1]. Although some NMD are acquired, the vast majority are genetic, and may display autosomal recessive, autosomal dominant, or X-linked inheritance [2]. It is also apparent that NMD phenotypes may vary widely even in individuals carrying the same disease-causing mutation, suggesting the existence of additional factors such as modifier genes that contribute to the onset, severity, and duration of the disease [3].

Recently, whole exome sequence analysis has identified two compound heterozygous recessive missense mutations in the gene *CACNA1H* in a patient with ALS [4,5]. *CACNA1H* encodes for Ca_v_3.2 channel, a member of the voltage-gated calcium channel family [6]. Ca_v_3.2 channels are widely expressed throughout the body including the central and peripheral nervous system, heart, kidney, smooth muscle, as well as in several neuroendocrine organs [7]. Through their ability to support low-threshold calcium influx (T-type current), they serve essential physiological processes including neuronal firing, hormone secretion, smooth muscle contraction, and myoblast fusion [8]. Their physiological implication is further exemplified by the existence of polymorphisms in *CACNA1H* associated with a number of human disorders including several forms of epilepsy [9], autism spectrum disorders [10,11], congenital pain [12], primary aldosteronism [13,14], and ALS [4,5].

In the present study, we report a patient with severe congenital amyotrophy in whom two compound heterozygous variants in *CACNA1H* were identified. Functional analysis of Ca_v_3.2 variants revealed altered channel gating, strengthening the genetic association of *CACNA1H* with NMD.

## Materials and methods

### Whole exome sequencing

Whole exome sequencing was performed at a commercial laboratory (GeneDx). Using genomic DNA from the proband and both parents, exonic regions and flanking splice junctions were selected, sequenced, and analyzed as per established proprietary protocols using an Illumina sequencing system with 100 bp or greater paired end reads. Reads were aligned to human genome build GRCh37/UCSC hg19 and analyzed for sequence variants using a custom‐developed analysis tool (Xome Analyzer). Mean depth of coverage was 100x.

### Plasmid cDNA constructs

The human wild-type Ca_v_3.2 in pcDNA3.1 [15] was used as template to introduce separately the V681L and D1233H mutations by site-directed mutagenesis using the Q5® Site-Directed Mutagenesis Kit (NEB) and the following pairs of primers: p.V681L: 5‘-GGGCCTCAGTttgCCCTGCCC-3‘ (forward) and 5‘-GACAGATGGCCAGGGGCC-3‘ (reverse); p.D1233H: 5‘-CCTGCGCATCcacAGCCACCG-3‘ (forward) and 5‘-AAGAAGTCGCTGGGCAGG-3‘ (reverse). Final constructs were verified by sequencing.

### Cell culture and heterologous expression

Human embryonic kidney tsA-201 cells were grown in DMEM medium supplemented with 10% fetal bovine serum and 1% penicillin/streptomycin (all media purchased from Invitrogen) and maintained under standard conditions at 37°C in a humidified atmosphere containing 5% CO_2_. Heterologous expression of Ca_v_3.2 channels was performed by transfecting cells with plasmid cDNAs encoding for Ca_v_3.2 channel variants using the calcium/phosphate method as previously described [16].

## Recording of T-type currents

Patch clamp recording of T-type currents in tsA-201 cells expressing Ca_v_3.2 channels was performed 72 h after transfection in the whole-cell configuration at room temperature (22–24°C). The bath solution contained (in millimolar): 5 BaCl2, 5 KCl, 1 MgCl2, 128 NaCl, 10 TEA-Cl, 10 D-glucose, 10 4-(2-hydroxyethyl)-1-piperazineethanesulfonic acid (HEPES) (pH 7.2 with NaOH). Patch pipettes were filled with a solution containing (in millimolar): 110 CsCl, 3 Mg-ATP, 0.5 Na-GTP, 2.5 gCl2, 5 D-glucose, 10 EGTA, and 10 HEPES (pH 7.4 with CsOH), and had a resistance of 2–4 MΩ. Recordings were performed using an Axopatch 200B amplifier (Axon Instruments) and acquisition and analysis were performed using pClamp 10 and Clampfit 10software, respectively (Axon Instruments). The linear leak component of the current was corrected online, and current traces were digitized at 10 kHz and filtered at 2 kHz. The voltage dependence of activation of Ca_v_3.2 channels was determined by measuring the peak T-type current amplitude in response to 150 ms depolarizing steps to various potentials applied every 10 s from a holding membrane potential of −100 mV. The current-voltage relationship (I/V) curve was fitted with the following modified Boltzmann Equation (1):

(1) $$I\left(V\right)=Gmax\frac{\left(V-Vrev\right)}{1+exp\frac{\left(V0.5-V\right)}{k}}$$

with *I*(*V*) being the peak current amplitude at the command potential *V, G*max the maximum conductance, *V*rev the reversal potential, *V*_0.5_ the half-activation potential, and *k* the slope factor. The voltage dependence of the whole-cell Ba^2+^ conductance was calculated using the following modified Boltzmann Equation (2):

(2) $$G\left(V\right)=\frac{Gmax}{1+exp\frac{(\left(V0.5-V\right)}{k}}$$

with *G*(*V*) being the Ba^2+^ conductance at the command potential *V*.

The voltage dependence of the steady-state inactivation of Ca_v_3.2 channels was determined by measuring the peak T-type current amplitude in response to a 150 ms depolarizing step to −20 mV applied after a 5 s-long conditioning prepulse ranging from −120 mV to −30 mV. The current amplitude obtained during each test pulse was normalized to the maximal current amplitude and plotted as a function of the prepulse potential. The voltage dependence of the steady-state inactivation was fitted with the following two-state Boltzmann function (3):

(3) $$I\left(V\right)=\frac{Imax}{1+exp\frac{\left(V-V0.5\right)}{k}}$$

with *I*_max_ corresponding to the maximal peak current amplitude and *V*_0.5_ to the half-inactivation voltage.

The recovery from inactivation was assessed using a double-pulse protocol from a holding potential of −100 mV. The cell membrane was depolarized for 2 s at 0 mV (inactivating prepulse) to ensure complete inactivation of the channel, and then to −20 mV for 150 ms (test pulse) after an increasing time period (interpulse) ranging between 0.1 ms and 2 s at −100 mV. The peak current from the test pulse was plotted as a ratio of the maximum prepulse current versus interpulse interval. The data were fitted with the following single-exponential function (4):

(4) $$\frac{I}{Imax}=A\times \left(1-exp\frac{-t}{\tau }\right)$$

where τ the time constant of channel recovery from inactivation.

### Statistical analysis

Values are presented as mean ± S.E.M. for *n* experiments. Statistical significance was determined using a one-way ANOVA test followed by Dunnett multiple comparison test. * *p*< 0.05, ** *p*< 0.005, *** *p*< 0.001.

## Results

### Clinical characteristics and genetic analysis

**Figure 1.**
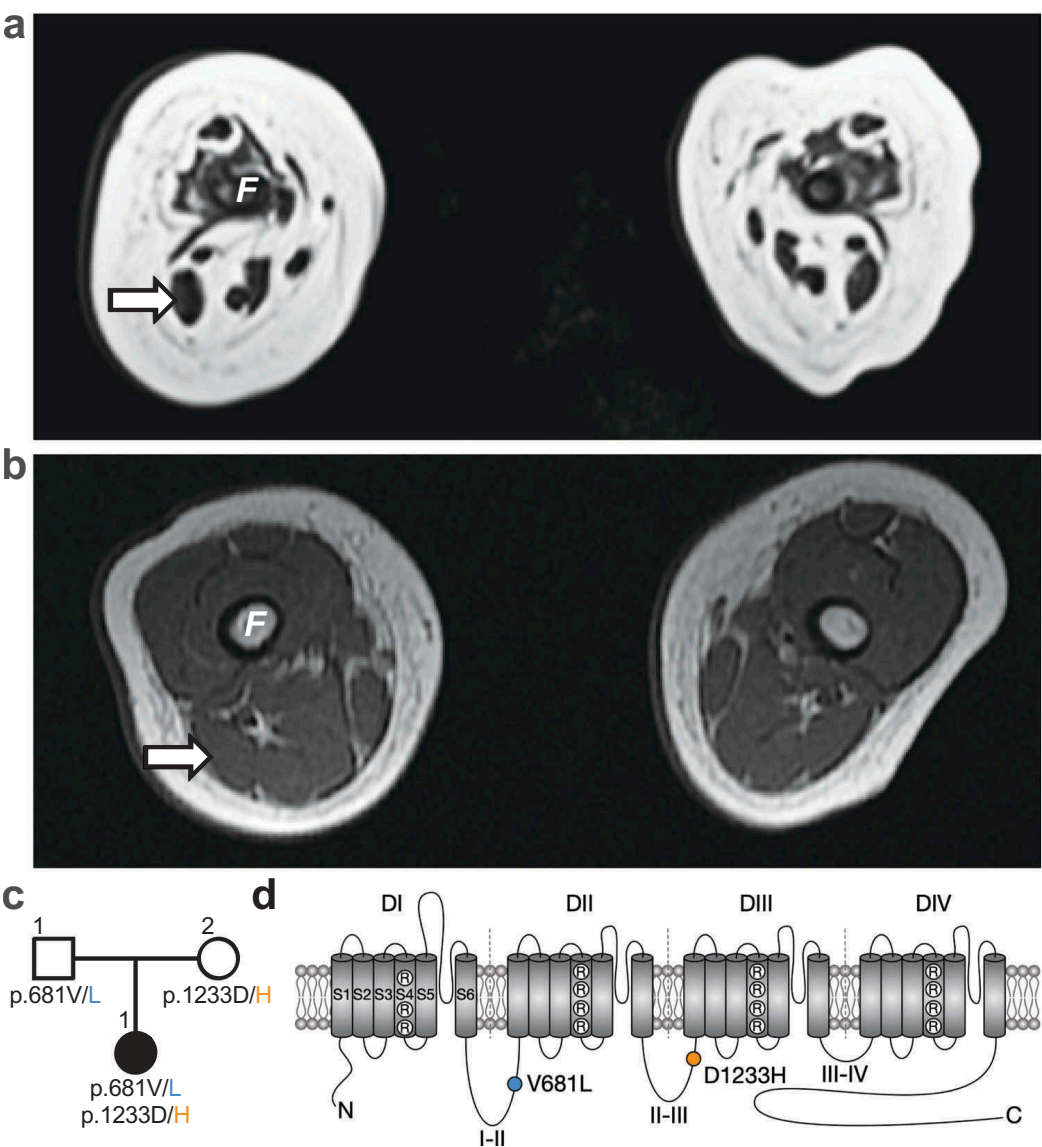
Whole exome sequencing identifies two *CACNA1H* missense mutations associated with severe congenital amyotrophy. (a) Whole-body magnetic resonance T1 weighted images (T1W1) 3T (axial view) of the thigh at 6 weeks old revealed severe muscle amyotrophy. No muscle was visualized in the anterior compartment. Her right biceps femoris (arrow) measured 8 × 5 mm at its widest. (b) For comparison, a T1WI (axial view) from a hypotonic boy with nemaline myopathy was performed at 4 months old. His biceps femoris measured 14 × 9 mm. *F*: femur. (c) Family pedigree chart. Filled and open symbols indicate affected and unaffected individuals, respectively. (d) Location of the p.V681L (blue circle) and p.D1233H mutations (orange circle) within the secondary membrane topology of Ca_v_3.2 channel.

The patient is a 13-month-old girl who was born at term following a pregnancy complicated by severe polyhydramnios treated with amnioreduction at 31 weeks. Her birth weight was 2.9 kg (20%ile). Her Apgar score (Appearance, Pulse, Grimace, Activity, and Respiration) used to summarize the health of newborn children was critically low (2 at 1-min and 5-min) and was intubated for poor respiratory effort, absent gag and suck. She had arthrogryposis, areflexia, and no antigravity movements. Nerve conduction studies showed intact sensory but absent motor responses. Magnetic resonance imaging (MRI) of her brain was unremarkable. However, the MRI of her muscles noted severe and diffuse amyoplasia with largest muscles being right biceps femoris (8 x 5 mm at widest diameter) and medial gastrocnemius (8 x 3 mm) (Figure 1(a,b)). A biopsy of the vastus lateralis revealed no viable muscle and repeat biopsies of the gastrocnemius noted a strong predominance of type 1 fibers and a marked variability of fiber size. She remains fully ventilated via tracheostomy with no antigravity movement at 13 months old. Whole exome sequencing identified two compound heterozygous variants (c.2041G>T and c.3697G>C) in *CACNA1H*, causing the substitution of the valine (V) 681 by a leucine (L) (p.V681L) and of the aspartic acid (D) 1233 by a histidine (H) (p.D1233H) in the Ca_v_3.2 T-type calcium channel, respectively. These mutations were inherited from the father and mother, respectively, who have no symptoms of muscle disease (Figure 1(c)). The p.V681L and p.D1233H mutations are located within the intracellular loops connecting domains I-II and domains II-III of Ca_v_3.2 channel, respectively (Figure 1(d)).

### Expression of Ca_v_3.2 channel variants

**Figure 2.**
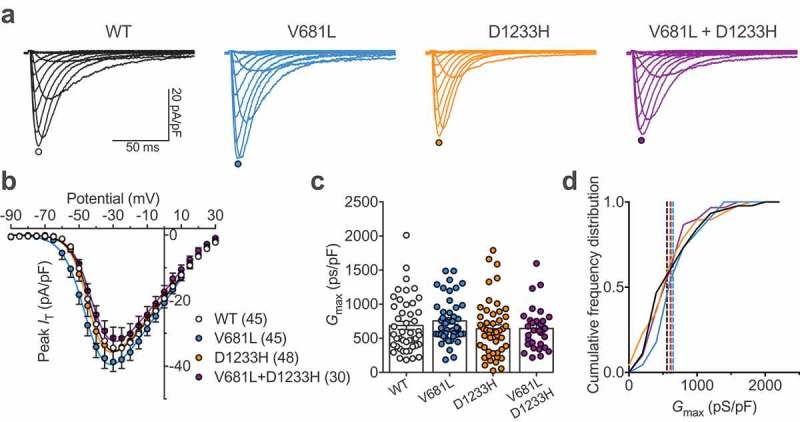
Functional expression of Ca_v_3.2 variants. (a) Representative T-type current traces recorded from cells expressing WT (black traces), V681L (blue traces), D1233H (orange traces), and co-expressing V681L and D1233H channel variants (purple traces) in response to 150 ms depolarizing steps ranging between −90 mV and 30 mV from a holding potential of −100 mV. (b) Corresponding mean peak current-voltage (*I*/*V*) relationship. (c) Corresponding mean maximal macroscopic conductance (*G*_max_) values obtained from the fit of the *I*/*V* curves with the modified Boltzmann Equation (1). (d) Cumulative frequency plot of data shown in (C). Dotted lines indicate the median values for each distribution (WT: 558 pS/pF; V681L: 648 pS/pF; D1233H: 601 pS/pF; V681L + D1233H: 615 pS/pF).

To assess the functional consequence of *CACNA1H* variants, the two mutations were introduced separately into the human Ca_v_3.2 channel, and functional expression was assessed by patch clamp recording of T-type currents in tsA-201 cells expressing Ca_v_3.2 channel variants. Cells expressing WT, V681L and D1233H Ca_v_3.2 channel variants, or co-expressing the two-channel variants (V681L + D1233H) to resemble the heterozygous status of the patient for the two mutations, displayed characteristic low-threshold voltage-activated T-type currents (Figure 2(a,b)). The maximal T-type conductance in cells expressing Ca_v_3.2 variants was similar (V681L: 756 ± 49 pS/pF, *n* = 45; D1233H: 640 ± 60 pS/pF, *n* = 48) and was not significantly different from cells expressing the WT channel (688 ± 59, *n* = 45) (Figure 2(c,d)) and Table (1)).

**Table 1.** Electrophysiological properties of human Ca_v_3.2 channel variants expressed in tsA-201 cells. Activation and inactivation kinetic values are shown for a depolarization step to −40 mV from a holding potential of −100 mV.

	Activation							Inactivation					RFI	
Ca_v_3.2 variant	V_0.5_ (mV)	*k*	(n)	τ (ms)	(n)	*G*_max_ (pS/pF)	(n)	V_0.5_ (mV)	*k*	(n)	τ (ms)	(n)	τ (ms)	(n)
WT	−39.9 ± 0.6	5.6 ± 0.2	45	5.8 ± 0.2	45	688 ± 59	45	−69.8 ± 0.6	4.4 ± 0.1	32	21.9 ± 1.0	32	377 ± 35	11
V681L	−43.4 ± 0.9**	5.4 ± 0.2	45	5.1 ± 0.3	45	756 ± 49	45	−75.3 ± 1.2***	4.4 ± 0.1	30	18.4 ± 0.9*	30	373 ± 29	18
D1233H	−41.1 ± 0.8	5.0 ± 0.2*	48	5.8 + 0.3	48	640 ± 60	48	−70.0 ± 0.7	4.1 ± 0.1	34	19.3 ± 1.1	34	343 ± 30	15
V681L+D1233H	−40.8 ± 0.6	5.6 ± 0.1	30	5.7 ± 0.2	30	644 ± 58	30	−74.0 ± 1.0**	4.7 ± 0.2	31	20.6 ± 0.8	31	435 ± 24	27

These results indicate that the V681L and D1233H mutations do not alter the expression level of Ca_v_3.2 channels.

### Gating properties of Ca_v_3.2 channel variants

**Figure 3.**
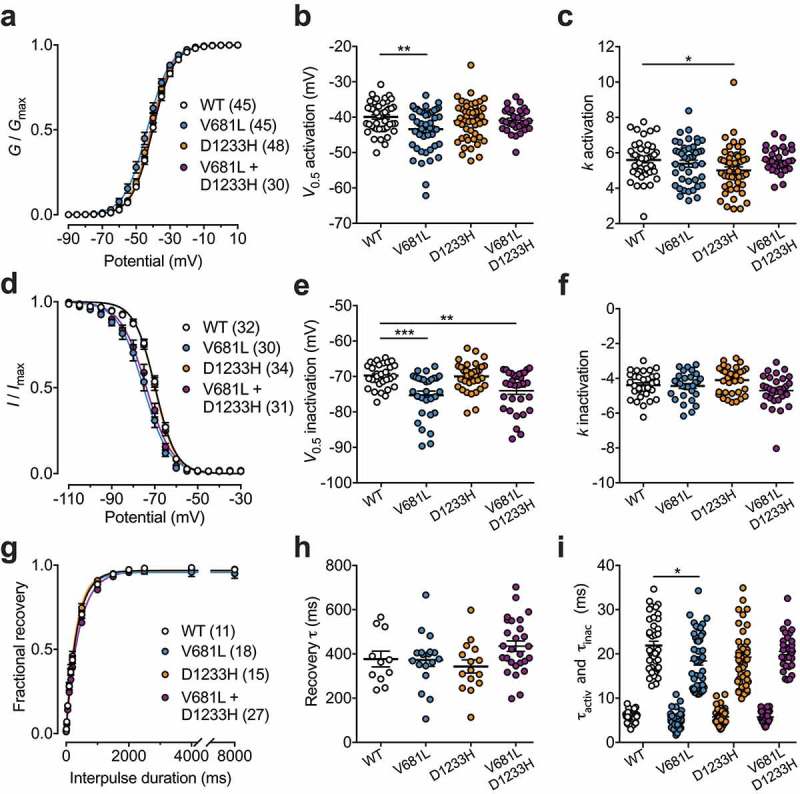
Electrophysiological properties of Ca_v_3.2 variants. (a) Mean normalized voltage-dependence of T-type current activation for cells expressing WT (white circles), V681L (blue circles), D1233H (orange circles), and co-expressing V681L and D1233H channel variants (purple circles). (b) Corresponding mean half-activation potential values obtained from the fit of the activation curves with the modified Boltzmann Equation (2). (c) Corresponding mean activation slope factor. (d) Mean normalized voltage-dependence of steady-state inactivation. (e) Corresponding mean half-inactivation potential values obtained from the fit of the inactivation curves with the two-state Boltzmann function (3). (f) Corresponding mean inactivation slope factor. (g) Mean normalized recovery from inactivation kinetics. (h) Corresponding mean time constant τ values of recovery from inactivation obtained by fitting recovery curves with the single-exponential function (4). (i) Mean time constant τ values of activation and inactivation of T-type currents obtained by fitting the rising and decay phase of the current with a single exponential function, respectively.

To assess the functional properties of Ca_v_3.2 variants, we analyzed the voltage-dependence of T-type current activation in cells expressing WT, V681L, D1233H, or co-expressing the V681L and D1233H channel variants (co-expression of the two separate cDNA constructs each carrying one mutation). The mean half-activation potential in cells expressing the V681L variant was shifted by 3.5 mV (*p* = 0.0039) toward hyperpolarized potentials (−43.4 ± 0.9 mV, *n* = 45) compared to cells expressing the WT channel (−39.9 ± 0.6 mV, *n* = 45) (Figure 3(a,b) and Table (1)). Although the mean half-activation potential of T-type currents measured in cells expressing the D1233H variant was not significantly altered, we observed a significant decrease (*p* = 0.0429) of the inactivation slope factor (5.0 ± 0.2, *n* = 48) compared to WT channels (5.6 ± 0.2, *n* = 45) (Figure 3(c) and Table (1)). To further investigate the gating properties of Ca_v_3.2 variants, we assessed the voltage-dependence of the steady-state inactivation of the T-type current. We observed a hyperpolarized shift of the mean half-inactivation potential by 5.5 mV (*p* < 0.0001) in cells expressing the V681L variant alone (−75.3 ± 1.2 mV, *n* = 30) and by 4.2 mV (*p* = 0.0027) in cells co-expressing the V681L and D1233H variants (−74.0 ± 1.0 mV, *n* = 31) compared to cells expressing the WT channel (−69.8 ± 0.6 mV, *n* = 32) (Figure 3(d–f) and Table (1)). In contrast, the voltage-dependence of steady-state inactivation of the T-type current in cells expressing the D1233H remained unaltered (Figure 3(d–f) and Table (1)). In addition, recovery from inactivation (Figure 3(g,h) and Table (1)) as well as inactivation and inactivation kinetics remain similar between the channel variants, and only a mild acceleration of the inactivation kinetic of the V681L variant (18.4 ± 0.9, *n* = 30) was observed compared to the WT channel (21.9 ± 1.0, n = 45) (Figure 3(i) and Table (1)).

Collectively, these data indicate that the V681L and D1233H mutations produce mild but significant alterations of the gating of Ca_v_3.2 by altering the voltage dependence of activation and inactivation of the channel.

### Window current of Ca_v_3.2 channel variants

**Figure 4.**
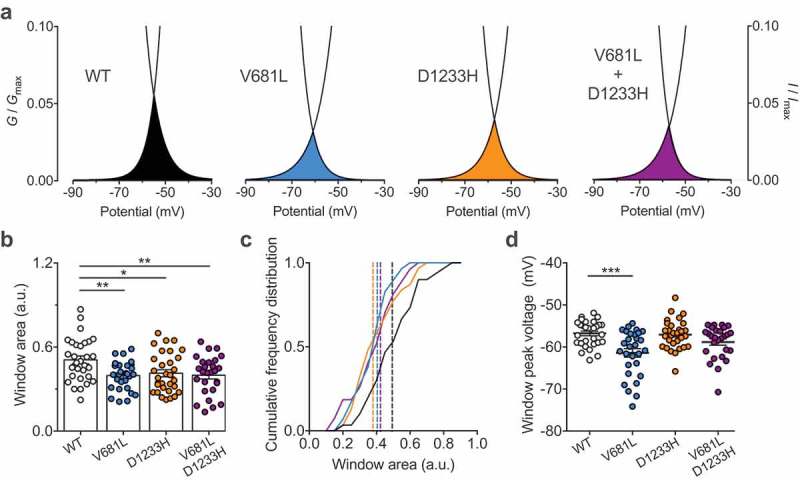
Window current of Ca_v_3.2 variants. (a) Representative activation (*G*/*G*_max_) – inactivation curves (*I*/*I*_max_) showing the window current for cells expressing WT (black), V681L (blue), D1233H (orange), and co-expressing V681L and D1233H channel variants (purple). (b) Corresponding mean window current measured as the area of the overlap between the activation and inactivation curves. (c) Corresponding frequency plot for each distribution. The dotted lines indicate the median values (WT: 0.4936; V681L: 0.4047; D1233H: 0.3792; V681L + D1233H: 0.4233). (D) Corresponding mean peak-voltage of the window currents. (d) Corresponding mean peak-voltage of the window currents.

Considering the effect of the V681L and D1233H mutations on the voltage dependence of activation and inactivation of Ca_v_3.2 channels, we analyzed the window current of Ca_v_3.2 variants as the area under the overlap between the activation and inactivation curves (Figure 4(a)). We observed a decrease of the window current generated by Ca_v_3.2 variants. For instance, the mean area of the window current was decreased by 22% (*p* = 0.0055) and 19% (*p* = 0.0188) in cells expressing the V681L and D1233H variants, respectively (Figure 4(b,c)). Consistent with this observation, the window current in cells co-expressing the V681L and D1233H channel variants were decreased by 22% (*p* = 0.0079) compared to cells expressing the WT channel (Figure 4(b,c)). In addition, we observed a hyperpolarized shift of the peak-voltage of the window current by 4.6 mV (*p* < 0.0001) in cells expressing the V681L variant (Figure 4(d)).

Altogether, these data indicate that alteration of the voltage-dependence of activation and inactivation caused by the V681L and D1233H mutations ultimately translates into a decrease of the window current supported by Ca_v_3.2 variants.

## Discussion

Here we describe two compounds heterozygous *CACNA1H* mutations identified in a case-unaffected-parents trio with infantile-onset NMD. The two mutations, V681L and D1233H, are located within the intracellular loops connecting domains I and II (I-II loop) and II and III (II-III loop) of the Ca_v_3.2 T-type calcium channel. Previous structure-function studies have documented the role of the I-II loop of Ca_v_3.2 in the control of the gating and surface expression of the channel [17,18]. Additionally, several human mutations associated with idiopathic generalized epilepsy and located within the I-II loop and II-III-loop of Ca_v_3.2 alter channel gating [19–22]. Consistent with these observations, our functional analysis of Ca_v_3.2 missense variants revealed several alterations of the gating properties of the channel. For instance, the V681L mutation produced a hyperpolarized shift of the voltage dependence of activation, as well as a concomitant hyperpolarized shift of the voltage dependence of inactivation. From a mechanistic viewpoint, this apparent mix gain- and loss-of-channel function can be explained by a model in which the V681L mutation may destabilize the closed state of the channel. This notion has recently been proposed for Ca_v_3.3 channels [23] and is consistent with the observation that T-type channels inactivate from both open and closed states [24,25]. Of particular importance is the observation that as a result of the effect on the voltage-dependence of activation and inactivation, the V681L mutation caused a marked decrease of the window current supported by Ca_v_3.2 channel. Likewise, decrease of the window current was also observed with the D1233H as a result of the increased slope of the activation curve without alteration of the mid-voltage.

**Figure 5.**
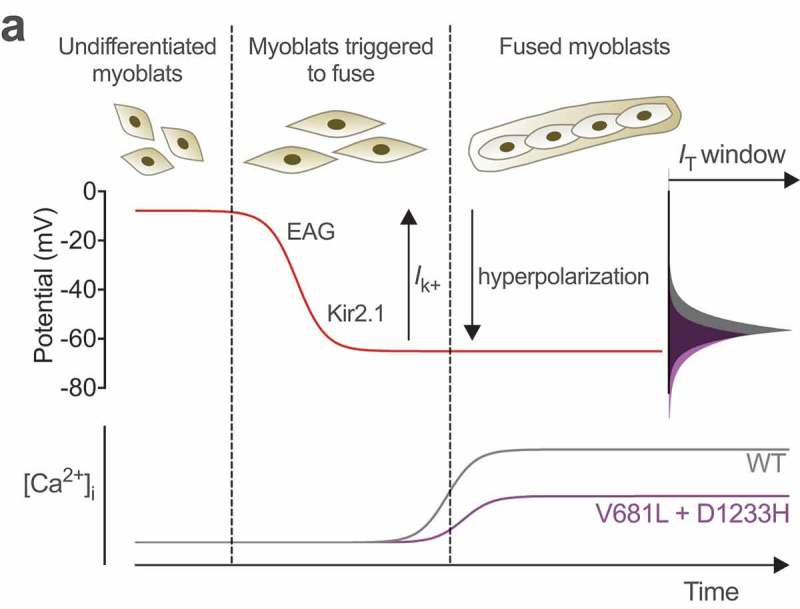
Hypothetical model for altered myogenesis by Ca_v_3.2 variants. While the membrane potential of undifferentiated myoblast is about −8 mV, sequential expression of the slow-inactivating voltage-gated ether-a-go-go (EAG) and inward-rectifying Kir2.1 potassium channels brings the membrane potential to approximately −65 mV (red line), allowing window calcium influx through Ca_v_3.2 channels (grey line) required for the fusion of myoblasts. Reduced window current caused the by V681L and D1233H mutations results in a decreased window calcium influx (purple line) which potentially compromises early stage myogenesis. Adapted from [30].

The question then arises as to how channel defects caused by the V681L and D1233H mutations may contribute to the disease phenotype. Alteration of the window current appears particularly relevant as an underlying mechanism of the amyotrophy observed in the proband for several reasons. First, Ca_v_3.2 channels are functionally expressed in embryonic skeletal muscle fibers where they present a developmental expression pattern consistent with a role in early-stage muscle differentiation. For instance, the T-type current density in muscle fibers increases transiently during prenatal myogenesis to reach a maximum expression at embryonic day E16 before to decrease dramatically until birth [26]. Second, extracellular calcium is required for the fusion of myoblasts [27] and a rise in intracellular calcium level precedes fusion [28,29]. Third, it was proposed that rise in intracellular calcium levels occurs via the window current supported by Ca_v_3.2 channels in response to the hyperpolarization of the plasma membrane that proceeds the fusion of the myoblasts [30]. Therefore, our observation that the p.V681L and p.D1233H mutations caused a reduction of the window current could have an important consequence at early stages of muscle differentiation and may represent an underlying mechanism of infantile-onset amyotrophy (Figure 5(a)). Additionally, several studies have reported the expression of T-type channels in motor neurons [31,32]. Although the role of T-type channels in motor neurons has not been investigated yet, it is likely that they contribute to some forms of neuronal excitability. Therefore, alteration of T-type channel activity is likely to affect muscle afferent signaling which may participate to some extend to motor symptoms.

In summary, we identified new *CACNA1H* variants in an individual with infantile-onset NMD. Clinical, histopathological, and electrophysiological findings indicating a loss-of-Ca_v_3.2 channel function suggest that severe congenital amyoplasia may be related to *CACNA1H*, which add to the growing list of *CACNA1H* channelopathies and would represent a new phenotype associated with mutations in this gene.

## References

[1] Zatz M, Passos-Bueno MR, Vainzof M. Neuromuscular disorders: genes, genetic counseling and therapeutic trials. Genet Mol Biol. 2016;39(3):339–348. 27575431 10.1590/1678-4685-GMB-2016-0019 PMC5004840

[2] Laing NG. Genetics of neuromuscular disorders. Crit Rev Clin Lab Sci. 2012;49(2):33–48. 22468856 10.3109/10408363.2012.658906

[3] Lamar KM, McNally EM Genetic modifiers for neuromuscular diseases. J Neuromuscul Dis. 2014;1(1):3–13. 25729645 10.3233/JND-140023 PMC4339951

[4] Steinberg KM, Yu B, Koboldt DC, et al Exome sequencing of case-unaffected-parents trios reveals recessive and de novo genetic variants in sporadic ALS. Sci Rep. 2015;5:9124. 25773295 10.1038/srep09124 PMC4360641

[5] Rzhepetskyy Y, Lazniewska J, Blesneac I, et al CACNA1H missense mutations associated with amyotrophic lateral sclerosis alter Cav3.2 T-type calcium channel activity and reticular thalamic neuron firing. Channels (Austin). 2016;466–477. 27331657 10.1080/19336950.2016.1204497 PMC5034776

[6] Weiss N, Zamponi GW T-type calcium channels: from molecule to therapeutic opportunities. Int J Biochem Cell Biol. 2019;108:34–39. 30648620 10.1016/j.biocel.2019.01.008

[7] Perez-Reyes E Molecular physiology of low-voltage-activated t-type calcium channels. Physiol Rev. 2003;83(1):117–161. 12506128 10.1152/physrev.00018.2002

[8] Zamponi GW, Striessnig J, Koschak A, et al The physiology, pathology, and pharmacology of voltage-gated calcium channels and their future therapeutic potential. Pharmacol Rev. 2015;67(4):821–870. 26362469 10.1124/pr.114.009654 PMC4630564

[9] Gambardella A, Labate A The role of calcium channel mutations in human epilepsy. Prog Brain Res. 2014;213:87–96. 25194484 10.1016/B978-0-444-63326-2.00004-1

[10] Splawski I, Yoo DS, Stotz SC, et al CACNA1H mutations in autism spectrum disorders. J Biol Chem. 2006;281(31):22085–22091. 16754686 10.1074/jbc.M603316200

[11] Lu AT, Dai X, Martinez-Agosto JA, et al Support for calcium channel gene defects in autism spectrum disorders. Mol Autism. 2012;3(1):18. 23241247 10.1186/2040-2392-3-18 PMC3558437

[12] Souza IA, Gandini MA, Wan MM, et al Two heterozygous Cav3.2 channel mutations in a pediatric chronic pain patient: recording condition-dependent biophysical effects. Pflugers Arch. 2016;468(4):635–642. 26706850 10.1007/s00424-015-1776-3

[13] Scholl UI, Stölting G, Nelson-Williams C, et al Recurrent gain of function mutation in calcium channel CACNA1H causes early-onset hypertension with primary aldosteronism. Elife. 2015;4:e06315. 25907736 10.7554/eLife.06315 PMC4408447

[14] Daniil G, Fernandes-Rosa FL, Chemin J, et al CACNA1H mutations are associated with different forms of primary aldosteronism. EBioMedicine. 2016;13:225–236. 27729216 10.1016/j.ebiom.2016.10.002 PMC5264314

[15] Dubel SJ, Altier C, Chaumont S, et al Plasma membrane expression of T-type calcium channel alpha(1) subunits is modulated by high voltage-activated auxiliary subunits. J Biol Chem. 2004;279(28):29263–29269. 15123697 10.1074/jbc.M313450200

[16] Proft J, Rzhepetskyy Y, Lazniewska J, et al The Cacna1h mutation in the GAERS model of absence epilepsy enhances T-type Ca^2+^ currents by altering calnexin-dependent trafficking of Ca_v_3.2 channels. Sci Rep. 2017;7(1):11513. 28912545 10.1038/s41598-017-11591-5 PMC5599688

[17] Vitko I, Bidaud I, Arias JM, et al The I-II loop controls plasma membrane expression and gating of Ca(v)3.2 T-type Ca2+ channels: a paradigm for childhood absence epilepsy mutations. J Neurosci. 2007;27(2):322–330. 17215393 10.1523/JNEUROSCI.1817-06.2007 PMC6672065

[18] Arias-Olguín II, Vitko I, Fortuna M, et al Characterization of the gating brake in the I-II loop of Ca(v)3.2 T-type Ca(2+) channels. J Biol Chem. 2008;283(13):8136–8144. 18218623 10.1074/jbc.M708761200 PMC2276388

[19] Khosravani H, Altier C, Simms B, et al Gating effects of mutations in the Cav3.2 T-type calcium channel associated with childhood absence epilepsy. J Biol Chem. 2004;279(11):9681–9684. 14729682 10.1074/jbc.C400006200

[20] Khosravani H, Bladen C, Parker DB, et al Effects of Cav3.2 channel mutations linked to idiopathic generalized epilepsy. Ann Neurol. 2005;57(5):745–749. 15852375 10.1002/ana.20458

[21] Peloquin JB, Khosravani H, Barr W, et al Functional analysis of Ca3.2 T-type calcium channel mutations linked to childhood absence epilepsy. Epilepsia. 2006;47(3):655–658. 16529636 10.1111/j.1528-1167.2006.00482.x

[22] Eckle VS, Shcheglovitov A, Vitko I, et al Mechanisms by which a CACNA1H mutation in epilepsy patients increases seizure susceptibility. J Physiol. 2014;592(4):795–809. 24277868 10.1113/jphysiol.2013.264176 PMC3934715

[23] Jurkovicova-Tarabova B, Cmarko L, Rehak R, et al Identification of a molecular gating determinant within the carboxy terminal region of Ca_v_3.3 T-type channels. Mol Brain. 2019;34. 30961646 10.1186/s13041-019-0457-0 PMC6454634

[24] Frazier CJ, Serrano JR, George EG, et al Gating kinetics of the alpha1I T-type calcium channel. J Gen Physiol. 2001;12(1):457–470. 10.1085/jgp.118.5.457 PMC2233834 11696605

[25] Serrano JR, Perez-Reyes E, Jones SW State-dependent inactivation of the alpha1G T-type calcium channel. J Gen Physiol. 1999;114(2):185–201. 10435997 10.1085/jgp.114.2.185 PMC2230639

[26] Berthier C, Monteil A, Lory P, et al Alpha(1H) mRNA in single skeletal muscle fibres accounts for T-type calcium current transient expression during fetal development in mice. J Physiol. 2002;539(3):681–691. 11897840 10.1113/jphysiol.2001.013246 PMC2290181

[27] Wakelam MJ The fusion of myoblasts. Biochem J. 1985;228(1):1–12. 3890835 10.1042/bj2280001 PMC1144947

[28] Entwistle A, Zalin RJ, Bevan S, et al The control of chick myoblast fusion by ion channels operated by prostaglandins and acetylcholine. J Cell Biol. 1988;106(5):1693–1702. 2453519 10.1083/jcb.106.5.1693 PMC2115067

[29] Rapuano M, Ross AF, Prives J Opposing effects of calcium entry and phorbol esters on fusion of chick muscle cells. Dev Biol. 1989;134(2):271–278. 2472983 10.1016/0012-1606(89)90099-7

[30] Bijlenga P, Liu JH, Espinos E, et al T-type alpha 1H Ca2+ channels are involved in Ca2+ signaling during terminal differentiation (fusion) of human myoblasts. Proc Natl Acad Sci U S A. 2000;97(13):7627–7632. 10861024 10.1073/pnas.97.13.7627 PMC16596

[31] Canto-Bustos M, Loeza-Alcocer E, González-Ramírez R, et al Functional expression of T-type Ca2+ channels in spinal motoneurons of the adult turtle. PLoS One. 2014;9(9):e108187. 25255145 10.1371/journal.pone.0108187 PMC4177857

[32] Zhang Z, David G Stimulation-induced Ca(2+) influx at nodes of Ranvier in mouse peripheral motor axons. J Physiol. 2016;594(1):39–57. 26365250 10.1113/JP271207 PMC4704498

